# The Impact of Gene Duplication, Insertion, Deletion, Lateral Gene Transfer and Sequencing Error on Orthology Inference: A Simulation Study

**DOI:** 10.1371/journal.pone.0056925

**Published:** 2013-02-25

**Authors:** Daniel A. Dalquen, Adrian M. Altenhoff, Gaston H. Gonnet, Christophe Dessimoz

**Affiliations:** 1 Eldgenössische Technische Hochschule Zurich, Department of Computer Science, Zürich, Switzerland; 2 Swiss Institute of Bioinformatics, Zürich, Switzerland; 3 European Bioinformatics Institute, Hinxton, Cambridge, United Kingdom; Hebrew University at Jerusalem, The Alexander Silberman Institute of Life Sciences, Israel

## Abstract

The identification of orthologous genes, a prerequisite for numerous analyses in comparative and functional genomics, is commonly performed computationally from protein sequences. Several previous studies have compared the accuracy of orthology inference methods, but simulated data has not typically been considered in cross-method assessment studies. Yet, while dependent on model assumptions, simulation-based benchmarking offers unique advantages: contrary to empirical data, all aspects of simulated data are known with certainty. Furthermore, the flexibility of simulation makes it possible to investigate performance factors in isolation of one another.

Here, we use simulated data to dissect the performance of six methods for orthology inference available as standalone software packages (Inparanoid, OMA, OrthoInspector, OrthoMCL, QuartetS, SPIMAP) as well as two generic approaches (bidirectional best hit and reciprocal smallest distance). We investigate the impact of various evolutionary forces (gene duplication, insertion, deletion, and lateral gene transfer) and technological artefacts (ambiguous sequences) on orthology inference. We show that while gene duplication/loss and insertion/deletion are well handled by most methods (albeit for different trade-offs of precision and recall), lateral gene transfer disrupts all methods. As for ambiguous sequences, which might result from poor sequencing, assembly, or genome annotation, we show that they affect alignment score-based orthology methods more strongly than their distance-based counterparts.

## Introduction

Two genes occurring in different species are called orthologous if they evolved from a single gene in the last common ancestor, whereas paralogous genes arise by gene duplication [1]. Because of this ancestral relationship, orthologs represent the evolutionary history of species most accurately and are also often believed to be functionally most similar [2], [3]. The identification of orthologs is therefore an important step in most analyses in comparative genomics [4], [5].

As the exact evolutionary history of most current-day species is not well understood, studies in phylogenetics, function inference and other areas of comparative genomics have to rely on computational inference of orthology.

A variety of methods for orthology inference has been developed over the last decade [6]–[10], but validation of these methods is inherently difficult for the same reasons that lead to their development: the precise evolutionary history of almost all sequence data observed today is unknown. Nevertheless, several orthology benchmarking approaches have been proposed. Early attempts used conservation of functional aspects, such as gene expression, protein-protein interaction, or Gene Ontology annotations, as indicators of orthology [11], [12]. However, this approach is open to debate, as orthology is solely defined by the evolutionary history of the genes, and the relation between evolution and function is not straightforward [3]. To address this problem, tests of phylogenetic congruence between orthologs and reference species tree have been pursued [12]. A fundamentally different approach, latent class analysis, assumes a mathematical model of the relation among orthology inference methods in terms of their false-positive and false-negative rates, and estimates these rates from predictions on a common set of genes by maximum likelihood [13]. Finally, there has been interest in the community for defining reference datasets for benchmarking orthology inference methods [14], for instance using the Yeast Gene Order Browser as a source for highly curated datasets [15], or by building sets of “Gold standard” reconciled gene/species trees [16], [17].

Although simulation has been occasionally used to assess individual orthology inference methods [18], [19], none of the aforementioned cross-method assessment studies has conducted a benchmark based on simulated data. While benchmarks based on simulated data have shortcomings of their own — namely a lack of realism due to their reliance on simplifying models — they also offer unique advantages [20]. They provide a controlled environment where the true evolutionary relationships are known. By varying the parameters of a distinct part of the simulation, we can systematically test a method and gain deeper insights into its behaviour. Although results on simulated data should not be taken at face value, they do provide a baseline for the performance of a method. In this sense, analyses on simulated data mainly contribute negative results, pointing to where algorithms do not work well: If a program performs poorly on simulated data, it is unlikely to perform well on real data [20].

In part, the limited role of simulation-based benchmarking in orthology inference evaluation can be explained by the challenge of simulating genome evolution: at the very least, simulation needs to account for sequence-level evolutionary events (character substitution, insertion, deletion) and genome-level evolutionary events (gene duplication and loss, speciation). Preferably, to investigate more relevant scenarios, the simulator should also introduce further events known to affect real data, such as lateral gene transfer or sequencing artefacts. Recently, we have introduced a simulation package for genome evolution, *Artificial Life Framework* (ALF), which can produce all the types of evolutionary events listed above [21].

Here, we assess the accuracy of a set of well-established orthology inference programs in simulated datasets obtained through different evolutionary processes. We use data simulated with ALF to investigate how gene duplications, lateral gene transfer, varying insertion and deletion rates and sequencing errors affect results.

## Results and Discussion

We investigated the performance of several orthology inference pipelines in light of four types of evolutionary events, described in more detail in the following sections. We sought to evaluate all widely-used orthology inference pipelines available as standalone software packages (i.e. able to analyse custom data on the user's computer). These were Inparanoid [7], the Markov clustering approach OrthoMCL [10], OrthoInspector [8], QuartetS [9] and OMA [6], [22]. For OMA we looked at predictions for pairwise orthologous relations (“OMA pairs”) as well as two ortholog groupings (the strict “OMA groups”, which identifies cliques of orthologs, and “OMA Hierarchical Orthologous Groups (HOGs)”, which infers genes that have descended from a single gene within specific taxonomic ranges). To investigate the performance of tree-based orthology inference, we also included SPIMAP [19] in our analysis. As points of reference, we also computed orthology based on simple best bidirectional hits (BBH) and reciprocal shortest distance (RSD). We used data simulated with ALF and varied the rate for each evolutionary event separately.

One challenge in benchmarking different orthology inference methods is to identify a general and relevant base of comparison. Indeed, the output of methods varies greatly—some of them producing gene trees labelled with speciation and duplication nodes, others producing various types of orthologous groups (reviewed in [5]). Despite these differences, all of these representations can be reduced to pairwise orthologous relations. For labelled trees, the pairs of orthologs are implied by the speciation nodes (as the Cartesian product of their two children leafsets). For groups, the implied orthologous pairs depend on the particular definitions but are also straightforward to derive (see Methods). Hence, we use the pairwise orthologous relations implied by each method's output as basis of comparison. Of note, several previous comparative studies have used pairwise orthologs as “common denominator” [11], [12], [16], while others have attempted to compare methods based on groups [15], [17].

We simulated two classes of datasets. The first class was aimed to be bacteria-like, with sequence lengths drawn from the length distribution of protein sequences observed in proteobacteria and species trees sampled from the tree of 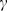-proteobacteria (see Methods). For the second class, we used a protein sequence length distribution that was close to that observed in mammals and sampled the species trees from the tree of mammals (see Methods). We accounted for variation in the performance of the methods caused by the underlying topology of the species tree by creating three parameter sets for each class, based on different species trees. Note that due to limitations in the implementation of SPIMAP, we could only evaluated it on the mammalia-like datasets (see Methods). Table 1 summarizes the baseline parameters and key statistics of all datasets. Results for the different parameter sets within the two classes were highly consistent, suggesting that our conclusions are not dependent on the precise shape of the species tree. For reasons of clarity, we therefore only show results for one parameter set of each class in the main text (see Figures S3, S4, S5, S6, S7 in File S1 for the other parameter sets).

**Table 1:** Baseline simulation parameters and key statistics.

	bacteria-like			mammalia-like		
	G1	G2	G3	M1	M2	M3
**parameters values**						
# of sequences	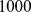					
distr. of seq. length	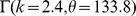			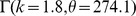		
min. sequence length	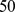					
substitution model	WAG					
insertion and deletion rate	0.000125					
# of species		30			20	
**key statistics**						
seq. length (mean, stdev)	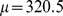 , 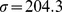			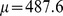 , 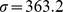		
avg. % gap chars in MSA	24.27	25.56	28.34	4.0	4.74	2.28
variance of % gap chars	58.0	52.6	52.4	12.6	15.5	7.5
total tree length	763.6	831.0	945.6	101.2	119.9	57.59
minimum tree height	31.70	41.80	46.59	14.70	11.55	7.693
maximum tree height	77.80	80.12	124.6	19.18	23.85	10.47
average tree height	41.36	55.70	62.64	17.48	14.79	8.996
average pairwise distance	72.60	92.31	90.07	14.50	16.80	8.74

Characteristics of the baseline parameters used to simulate the datasets and resulting key statistics for sequence length, insertions and deletions, and tree topology. Distances and tree height/length given in PAM units.

### Increasing the duplication rate shows different trade-offs between methods

To investigate the effect of the rate of gene duplication on orthology inference, we generated datasets with increasing duplication and loss rates and ran the different orthology inference methods (Table 2).

**Table 2:** Simulation parameters for analysis of gene duplication.

	bacteria-like			mammalia-like		
	G1	G2	G3	M1	M2	M3
**10% duplication**						
duplication/loss rate	0.003	0.002	0.0017	0.0065	0.0065	0.013
**20% duplication**						
duplication/loss rate	0.006	0.004	0.0035	0.013	0.016	0.025
**30% duplication**						
duplication/loss rate	0.0105	0.008	0.007	0.025	0.03	0.05
**40% duplication**						
duplication/loss rate	0.017	0.0125	0.0115	0.0455	0.055	0.09

Parameters for gene duplication and gene loss used to simulate the datasets for investigating the effect of gene duplication on orthology inference. These rates are per gene, per PAM unit (i.e. relative to substitutions).

**Figure 1:**
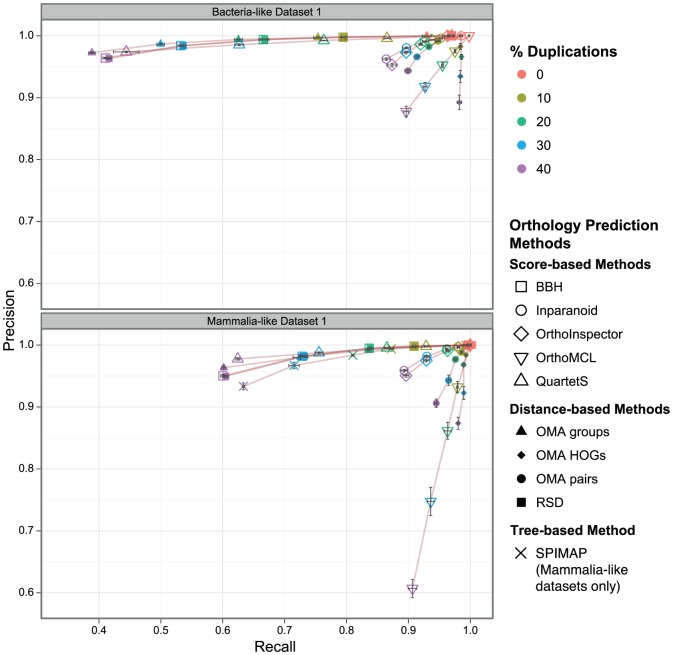
Orthology inference vs. gene duplication. Precision/recall of orthology inference with different proportions of genes with a history of duplications. Each data point corresponds to the mean over all orthologous relations in five replicates (with 95% confidence interval of the mean values in both dimensions).

For most methods, increasing the gene duplication rate affects recall (proportion of true orthologs that are detected) more than precision (proportion of predicted orthologs that are true). This effect is visualised in Figure 1, with recall on the x-axis and precision on the y-axis. More specifically, the different programs fall into three main groups. The first group comprises methods that keep a high precision even when duplication rates get higher, accepting a substantial drop in recall. QuartetS, the OMA groups, SPIMAP as well as simple best bidirectional hits (BBH) and reciprocal smallest distance (RSD) fall under this category. Of these methods, QuartetS appears to perform best overall, with higher recall at similar or better precision than the other methods in most scenarios. Only at high duplication rates, the tree-based method (SPIMAP) has the edge in terms of recall over the other methods of the group (see also Figure S3 in File S1). BBH and RSD are very similar with respect to each other. Both methods have a higher recall than the OMA groups, but precision suffers more when duplication rates are higher. For the mammalia-like datasets, which have less divergent sequences but higher duplication/loss rates, BBH/RSD perform almost identically to OMA groups.

Methods of the second category compromise between precision and recall, accepting a decrease in precision in order to control recall. This category includes Inparanoid, OrthoInspector and OMA pairs. Of the three, Inparanoid has generally the highest precision whereas OMA pairs has the highest recall.

OrthoMCL and OMA HOGs form the third category. They keep a high recall at the expense of precision when the number of duplications increases, with OrthoMCL suffering most in the mammalia-like dataset.

This behaviour can be viewed as indicating that all the methods are able to detect gene duplications, and shows the different choices of trade-off that the methods make when inference gets harder. While some methods lean toward a low false positive rate to recovering more orthology relations, others lean toward higher recall at the expense of precision. This finding is in line with our previous, smaller study [21].

**Figure 2:**
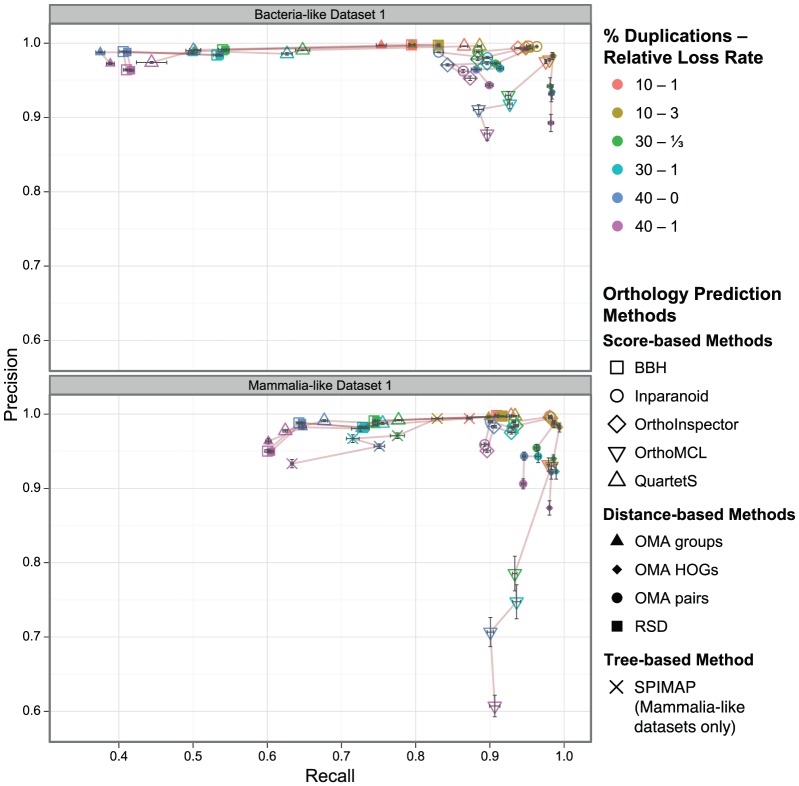
Orthology inference vs. gene duplication with varying loss rates. Precision/recall of orthology inference with different proportions of genes with a history of duplications and varying relative loss rates. Each data point corresponds to the mean over all orthologous relations in five replicates (with 95% confidence interval of the mean values in both dimensions).

To study the influence of duplication and loss rates separately, we simulated three scenarios where the gene loss rate was different from the rate of gene duplication (Table 3). Our analysis shows that a higher relative loss rate leads to a decrease in precision, in particular when the duplication rate is high (Figure 2). This behaviour could be explained by the increasing number of differential gene losses occurring at higher loss rates [23]. At the same time, change in recall is less consistent among methods. Some methods exhibit an decreased recall when the loss rate increases (QuartetS, SPIMAP). For OrthoMCL and OMA HOGs, recall stays roughly the same. BBH and RSD perform identically, with little change in recall for the bacteria-like datasets and an increased recall for mammalia-like datasets. Inparanoid, Orthoinspector and OMA pairs show a increase in recall on bacteria-like datasets and little change for the mammalia-like datasets. Recall for OMA groups increases with increasing relative loss rate on the bacteria-like datasets but increases on the mammalia-like datasets.

**Table 3:** Simulation parameters for analysis of gene duplication.

	bacteria-like			mammalia-like		
	G1	G2	G3	M1	M2	M3
**10% duplication, rel. loss rate = 1**						
duplication/loss rate	0.003	0.002	0.0017	0.0065	0.0065	0.013
**10% duplication, rel. loss rate = 3**						
duplication rate	0.0026	0.002	0.0017	0.0065	0.007	0.0122
loss rate	0.0078	0.006	0.0051	0.0195	0.021	0.0366
**30% duplication, rel. loss rate = ** 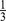						
duplication rate	0.0087	0.0066	0.00585	0.0201	0.0246	0.0399
loss rate	0.0029	0.0022	0.00195	0.0067	0.0082	0.0133
**30% duplication, rel. loss rate = 1**						
duplication/loss rate	0.0105	0.008	0.007	0.025	0.03	0.05
**40% duplication, rel. loss rate = 0**						
duplication rate	0.0125	0.0093	0.0083	0.03	0.035	0.0585
loss rate	0					
**40% duplication, rel. loss rate = 1**						
duplication/loss rate	0.017	0.0125	0.0115	0.0455	0.055	0.09

Parameters for gene duplication and gene loss used to simulate the datasets for investigating the effect of gene duplication on orthology inference. These rates are per gene, per PAM unit (i.e. relative to substitutions).

### Lateral gene transfer disrupts all orthology inference methods

To investigate the impact of lateral gene transfer (LGT) on orthology, we generated evolutionary scenarios with 10–80% of the genes within each species originating from an LGT event, replacing their ortholog in the recipient species (see Table 4 and Methods).

**Table 4:** Simulation parameters for analysis of LGT.

	bacteria-like			mammalia-like		
	G1	G2	G3	M1	M2	M3
**0% lateral gene transfer**						
duplication/loss rate	0.003	0.002	0.0017	0.0065	0.0065	0.013
**10% lateral gene transfer**						
duplication/loss rate	0.0025	0.0018	0.0017	0.0058	0.0068	0.011
LGT rate	0.0025	0.0018	0.0017	0.0058	0.0068	0.011
**20% lateral gene transfer**						
duplication/loss rate	0.0025	0.0018	0.0017	0.0058	0.0068	0.011
LGT rate	0.0045	0.0034	0.0031	0.0108	0.0136	0.022
**40% lateral gene transfer**						
duplication/loss rate	0.0025	0.0018	0.0017	0.0058	0.0068	0.011
LGT rate	0.0085	0.0064	0.0059	0.0215	0.025	0.0405
**60% lateral gene transfer**						
duplication/loss rate	0.0025	0.0018	0.0017	0.0058	0.0068	0.011
LGT rate	0.0125	0.0092	0.0087	0.032	0.038	0.063
**80% lateral gene transfer**						
duplication/loss rate	0.0025	0.0018	0.0017	0.0058	0.0068	0.011
LGT rate	0.0175	0.0128	0.0127	0.097	0.064	0.19

Parameters for gene duplication, gene loss and LGT used to simulate the datasets for investigating the effect of LGT on orthology inference. These rates are per gene, per PAM unit (i.e. relative to substitutions).

**Figure 3:**
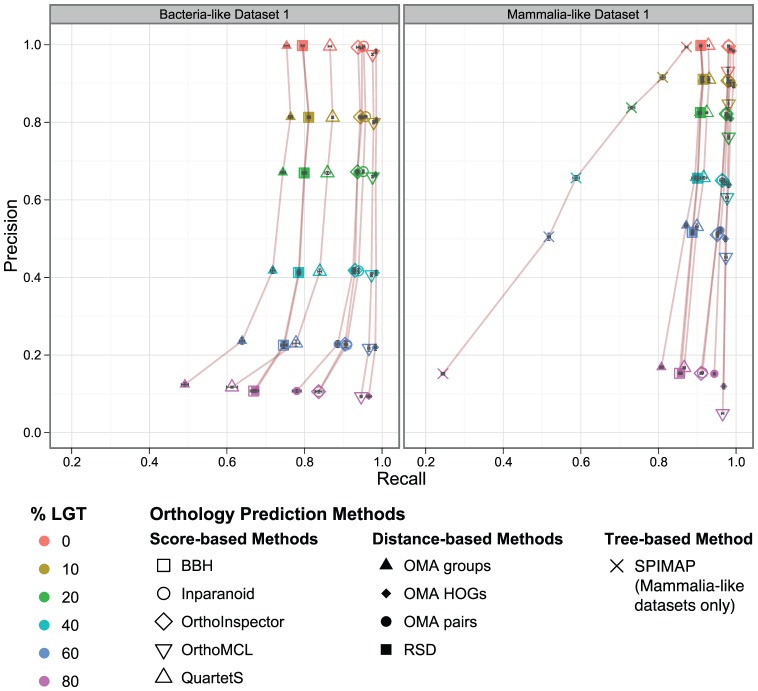
Orthology inference vs. LGT. Precision/recall of orthology predictions with different proportions of genes with a history of lateral gene transfer. Each data point corresponds to the mean over all orthologous relations in five replicates (with 95% confidence interval of the mean values in both dimensions).

The effect of LGT on orthology inference is very similar for all distance- and score-based methods: the more laterally transferred genes a dataset contains, the lower the precision (Figure 3). On the other hand, recall is mostly stable except for the datasets with the highest LGT rates. From this behaviour, it is apparent that all of the methods tested have trouble distinguishing laterally transferred genes from true orthologs.

Although it was recently shown that the environment plays an important role in the propensity of LGT, within each environment, the frequency of LGT is generally higher for closely related species [24]. To investigate the performance of orthology inference on evolutionary scenarios with smaller distances, we performed our analysis on data simulated on mammalia-like trees, that fulfill this characteristic.

While most of our findings for the bacteria-like datasets also apply to the mammalia-like datasets, the precision of OrthoMCL appears to be significantly lower than for the other methods in the mammalia-like datasets. The clear outlier in this analysis is tree-based SPIMAP, for which both precision and recall decrease as the proportion of laterally transferred genes increases. This behaviour suggests that the method is especially sensitive to the disruptive effect of LGT on tree inference and reconciliation.

Our results are not unexpected given the fact that none of the programs investigated incorporates a method for detecting LGT, but they underline a potential shortcoming of current methods for orthology detection: a lack of any mechanism to detect LGT during orthology inference could be particularly troubling, considering the prevalence of LGT in prokaryotic evolution.

### All methods handle insertions and deletions similarly well

**Figure 4:**
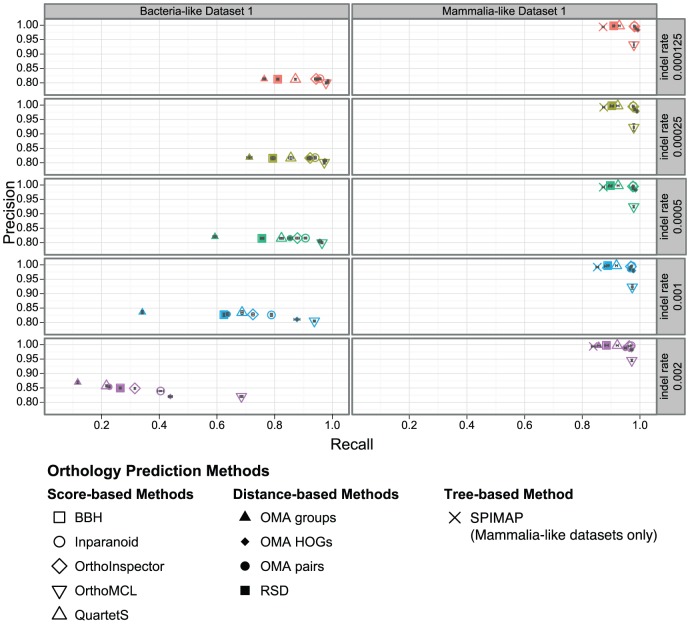
Orthology inference vs. insertions and deletions. Precision/recall of orthology predictions with different rates of insertion and deletion events. Each data point corresponds to the mean of over all orthologous relations in five replicates (with 95% confidence interval of the mean values in both dimensions).

Next, we investigated the impact of the insertion and deletion rate on orthology inference (Table 5). All methods proved to be robust to moderate levels of insertions and deletions (Figure 4). Precision is hardly affected — if anything, precision increases with increasing insertion and deletion rates.

**Table 5:** Simulation parameters for analysis of insertions and deletions.

	bacteria-like			mammalia-like		
	G1	G2	G3	M1	M2	M3
**parameters for all simulations**						
duplication/loss rate	0.0025	0.0018	0.0017	0.0065	0.0065	0.013
LGT rate	0.0025	0.0018	0.0017	0	0	0
**insertion and deletion rates**						
	0.00025					
	0.0005					
	0.001					
	0.002					

Parameters for gene duplication, gene loss, LGT, and insertions and deletions used to simulate the datasets for investigating the effect of insertions and deletions on orthology inference. These rates are per gene for duplication, loss and LGT, and per site for insertions and deletions, per PAM unit (i.e. relative to substitutions).

The primary effect of insertion and deletion can be observed on recall. As insertions and deletions lead to fewer homologous sites in the alignment, the programs have to base their predictions on less information. This characteristic makes orthology inference more difficult, which, as was the case for gene duplications, is reflected in the decrease of recall. The effect of higher rates is more pronounced with larger distances, as insertions and deletions can accumulate over a longer period of time. For small distances, there seems to be sufficient information for effective orthology inference, even for high insertion and deletion rates.

**Figure 5:**
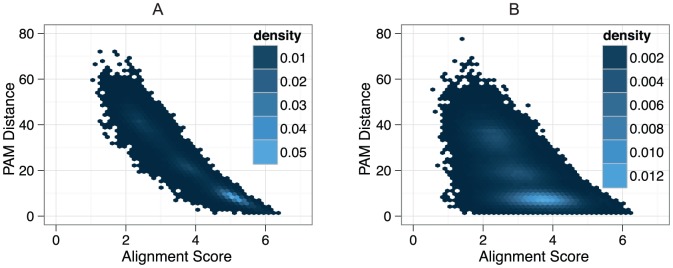
Alignment score vs. distance. Pairwise alignment scores compared to Percent Accepted Mutation (PAM) distance for one run of mammalia-like dataset 1. A) For insertion and deletion rate 0.001. Scores were normalised by the sum of the aligned characters in both sequences. 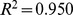; B) with 18 percent ambiguous characters. Scores were normalised by the sum of the aligned characters in both sequences. 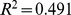.

Comparing BBH and RSD, one could expect that insertions and deletions would perturb alignment scores more strongly than distance estimation, because there is a roughly linear relation between alignment length and alignment score. This would give distance-based methods an edge over score-based methods. However, we observe virtually no difference in performance between BBH and RSD. In trying to investigate this phenomenon, we computed the Pearson correlation between score and distance in the presence of high insertion and deletion rates. With 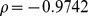, the very strong (negative) correlation between score and distance indicates that BBH and RSD are almost equivalent objectives in the context of high insertion and deletion rates (Figure 5A).

### Sequence artefacts tend to affect score-based methods more strongly than distance-based methods

Finally, we simulated datasets under increasingly high sequencing and assembly error rates, obtained by replacing randomly selected stretches of sequence with the ambiguity character X (see Methods section).

**Figure 6:**
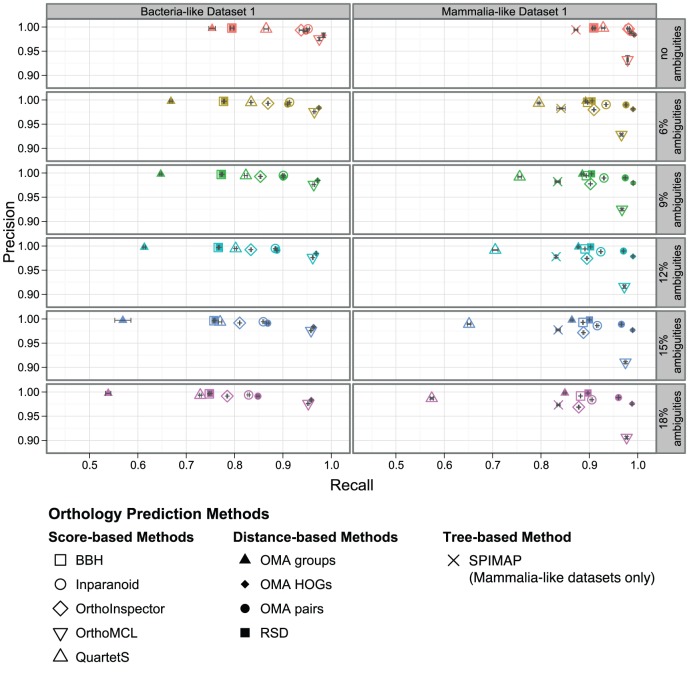
Orthology inference vs. sequencing artefacts. Precision/recall of orthology predictions with different proportions of ambiguous (i.e. “X”) characters. Each data point corresponds to the mean of over all orthologous relations in five replicates (with 95% confidence interval of the mean values in both dimensions).

As for insertions and deletions, the effect of sequencing errors is different for bacteria-like and mammalia-like datasets (figure 6). For bacteria-like datasets, mainly recall is affected, similarly to the behaviour we observed for insertions and deletions. There are slight differences between methods. In particular, while the performance of OrthoMCL, OMA HOGs, BBH and RSD hardly changes when sequencing errors are introduced, the other methods exhibit a stronger drop in recall. We observed the largest difference for OMA groups, followed by Inparanoid, OrthoInspector and QuartetS. For pairwise orthologs from OMA, the loss in recall is less pronounced.

For mammalia-like datasets on the other hand, there is a stronger deterioration with the increase of sequence artefacts, mainly in terms of recall, except for tree-based SPIMAP, where more artefacts instead lead to a slight decrease in precision (Figure 6, right column). Interestingly, we observed a small, but consistent difference between distance-based methods (Figure 6, filled symbols) and score-based methods (Figure 6, empty symbols). We could again reason that ambiguous characters perturb alignment scores more than distance estimates. But contrary to insertion/deletion processes, sequencing errors do not occur along the underlying evolutionary process, so score drops due to ambiguous characters are not correlated to evolutionary distance. We verified this hypothesis by plotting the alignment scores of all BBH pairs against their distance and computing the correlation of score and distance (Figure 5B). The correlation 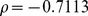 is considerably lower than for insertion and deletion, which explains the difference in performance between distance-based and score-based methods. The other unexpected result is the behaviour of QuartetS in the mammalia-like dataset: while the drop in precision is commensurate with other score-based methods, the drop in recall is much larger. To test whether this is primarily due to the difference in sequence length distribution between the two datasets, we simulated data based on mammalia-like parameters, but using the length distribution of the bacteria-like datasets. We observed a substantial increase in recall on these datasets (Figure S8 in File S1). This suggests that QuartetS struggles with sequencing and assembly artefacts on longer sequences, though other factors might also be at play.

## Conclusions and Outlook

In this study, we analysed the effect of different types of evolutionary events on some of the most common tools for orthology inference available as standalone software packages. Our results show that while some events are well handled by most methods, others have a detrimental effect on predictions. We observed that gene duplications and insertion/deletion events mainly affect recall—the proportion of correct orthologous pairs predicted by each method. Ambiguities in the sequences such as those that could arise through sequencing errors appeared to also mainly affect recall in the bacteria-like datasets, whereas in the mammalia-like datasets precision—the proportion of predicted orthologous pairs that are correct—was affected as well, particularly for those methods that rely on alignment scores for their clustering. According to our analysis, the reason for this behaviour is the higher robustness of distance estimation in light of sequencing errors compared to alignment scores.

We observed that LGT dramatically decreases precision for all methods analysed. Given the importance of LGT in prokaryotic evolution, an improvement of current orthology inference methods to cope with lateral gene transfer appears to be worth pursuing.

In terms of individual methods, this study confirms the broad observation of previous benchmarks on empirical data that most methods are situated on a “Pareto frontier” between precision and recall, with different methods making different trade-offs [11], [12]. In particular, and consistent with our analysis in [12], we do not observe a fundamental difference between tree-based and graph-based orthology inference methods in terms of prediction quality.

Overall, we hope to have convincingly shown that simulation-based orthology benchmarking can provide insights into the performance of orthology inference methods. We stress that all results thus obtained depend on the assumptions underlying the simulations; to which extent these generalize to real data necessarily will depend on the nature of these real data. Nevertheless, simulation-based benchmarks can provide specific hypotheses whose validity can be further investigated on empirical benchmarks.

## Materials and Methods

We used ALF [21] to create the datasets for our analysis. The following sections detail the parameters used for the different aspects of the simulation. Table 1 summarizes the baseline parameters and key statistics for all simulations. Parameter values that were varied in the different comparisons are summarised in Tables 2, 3, 4, and 5.

### Topologies

**Figure 7:**
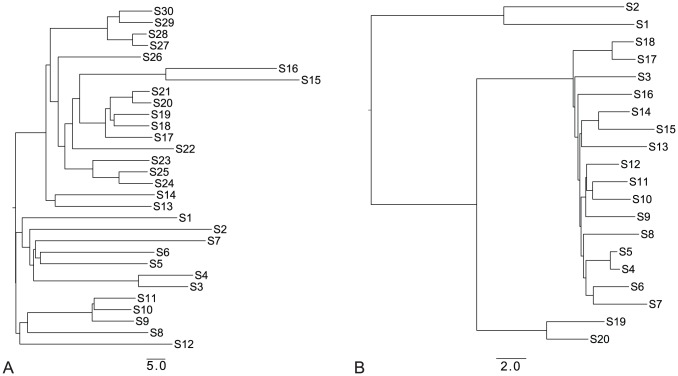
Species trees for bacteria-like dataset 1 and mammalia-like dataset 1. Species trees used in the simulations of bacteria-like dataset 1 (A), sampled from bacteria tree, and mammalia-like dataset 1 (B), sampled from mammalia tree.

We sampled trees from the tree of 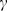-proteobacteria and from the tree of mammalia as estimated by the OMA project [22]. The bacteria tree consisted of 224 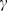-proteobacteria species. From these, we sampled 30 species. The mammalia tree contained of 37 species of which we sampled 20. In both cases we sampled three different topologies. All pairs of species were required to be separated by a distance of at least 1 Point Accepted Mutation (PAM) unit. Key statistics of the resulting trees are given in Table 1. The topologies are also provided in Figure 7 and in Figure S1 in File S1.

### General simulation setup and parameters

As we describe below, we tested a total of 27 different simulation scenarios. Each of these 27 analyses was performed on simulations derived from the 3 mammalia-like phylogenies/parameters and 3 bacteria-like phylogenies/parameters, and replicated in 5 synthetic evolution runs with ALF (using identical parameters). Thus, we generated a total of 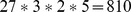 synthetic datasets, on which we ran the different orthology inference methods.

In all simulations, we used the WAG substitution model [25]. Substitution rates were kept constant within and among gene families. The ancestral (root) genomes consisted of 1000 sequences that were randomly sampled from the stationary distribution of the substitution model. Sequence lengths were sampled from a gamma distribution fitted on real data from bacterial and mammalian genomes, respectively (Table 1).

For all simulations unless otherwise stated, insertions and deletions were both set to occur at a rate of 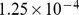 per PAM per site. The insertion and deletion length were sampled from a Zipfian distribution with exponent parameter 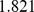 [26]. For each parameter set we repeated the simulation five times in order to get an idea of the variance within a parameter set.

### Simulations with varying gene duplication rates

For each topology we created datasets with four different proportions of genes with a duplication background, ranging from 10–40%. These proportions lie within the range that is believed to be present in real species [27]. In each duplication event, only single genes were duplicated. Gene loss rates were set equal to duplication rates in order to keep genome sizes roughly constant. Because ALF models gene duplications and losses as Markovian processes, the resulting gene families could result in multiple levels of nested duplication and losses (see Figure S2 in File S1 for an example gene tree). We empirically determined appropriate rates by checking the proportion of genes, that arose by gene duplication, across all resulting genomes of five simulation runs (Table 2).

In addition, we simulated three scenarios where the relative gene loss rates were different from the duplication rates. For the first scenario, the proportion of genes with a duplication background was 10% with a loss rate that was three times the duplication rate. For the second scenario, 30% of genes had a duplication background and the loss rate was set to a third of the duplication rate. In the third scenario, we set the loss rate to 0 and created datasets with 40% of genes having a duplication background.

### Simulations with varying lateral gene transfer rates

We simulated lateral gene transfer as orthologous replacements, i. e. transferred genes replaced their existing ortholog in the recipient species. While exact numbers are still debated, it has been argued that the cumulative effect of LGT could be as high as 80 percent [28], [29]. As with duplications, we therefore created datasets with different proportions of genes having undergone LGT, ranging from 10–80% (Table 4). Per event, only one gene was transferred. We also allowed 10% of gene duplications and losses in all datasets.

### Simulations with varying insertion and deletion rates

Starting from the default base insertion and deletion rates of 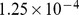 per PAM per site, we simulated datasets for four more parameter sets where we doubled insertion and deletion rates each time (Table 5).

### Simulations with sequence artefacts

For the simulation of sequencing and assembly errors, we followed the approach of [30]. They noticed that in low-coverage genomes, ambiguities made up on average between 9–15 percent of coding sequences and that the length of these stretches were normally distributed.

We used datasets with 10 percent duplication as a base case and in each gene of every genome substituted a randomly selected single stretch of amino acids with X characters. The length of each stretch was chosen by drawing a proportion from a normal distribution as described by [30] and multiplying it by the length of the sequence. To simulate different amounts of sequencing errors, we varied the mean of the length proportion 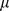 between 6 and 18%. The standard deviation of the length proportion was fixed to 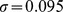 for all datasets.

### Comparison of orthology inference pipelines

We used six different orthology inference programs in our analysis. Inparanoid [7] computes pairwise orthologs, whereas OrthoMCL [10], QuartetS [9], OrthoInspector [8], SPIMAP [19] and OMA StandAlone (Dessimoz et al. [31], http://omabrowser.org/standalone) also implement clustering of gene families across multiple species. For OMA, we consider 3 different variants: 1) OMA pairs, which are pairs of orthologs obtained by the OMA algorithm [6]; 2) OMA Groups, a stringent clustering strategy based on cliques of OMA pairs [6]; and 3) OMA HOGs, hierarchical orthologous groups obtained using the GETHOGs algorithm [22], [32]. For SPIMAP, we used the clusters from OrthoMCL of size 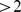 as initial groups and followed the pipeline described by Rasmussen and Kellis [19]. Because SPIMAP requires alignments of nucleotide sequences, we back-translated the simulated protein sequences into codon sequences, using a single codon per amino acid for each column of the alignment. Finally, we performed gene and species tree reconciliation on the inferred gene trees. Unfortunately, SPIMAP returned errors on the bacteria-like datasets, that we could not resolve. Therefore we only report results for the mammalia-like datasets. Additionally, we extracted plain BBH matches, based on Smith-Waterman alignment scores, and RSD matches from the all-vs-all phase of the OMA pipeline. We ran all tools with their default or recommended parameters. For tools with orthologous groups as output, we created the set of induced orthologous pairs by pairing each member of a group with all other members of that group belonging to a different species. For the tree-based method (SPIMAP) we took the Cartesian product of the two children leafsets of all speciation nodes in the reconciled tree. We then compared the set of predicted pairwise orthologs to the set of true orthologs as given by the simulation.

## Supporting Information

File S1 **PDF containing Figures S1-S8.**
(PDF)

Dataset S1 **ZIP archive with all parameter files used to generate the simulated datasets with ALF** [**21**] **.** The Fasta files for all 810 datasets can be downloaded from http://orthology.benchmarkservice.org/simdata/.
(ZIP)
