# Supplementary material for: The Impact of Gene Duplication, Insertion, Deletion, Lateral Gene Transfer and Sequencing Error on Orthology Inference: A Simulation Study
Source: PLoS One. 2013 Feb 25;8(2):e56925. doi: 10.1371/journal.pone.0056925 (PMC3581572; doi:10.1371/journal.pone.0056925)
Supplement: File S1 — PDF containing Figures S1-S8. (PDF) [file pone.0056925.s001.pdf]

Supplementary figures

The Impact of Gene Duplication, Insertion, Deletion, Lateral Gene Transfer and Sequencing Error on Orthology Inference — A Simulation Study

Dalquen et al.

Species Trees

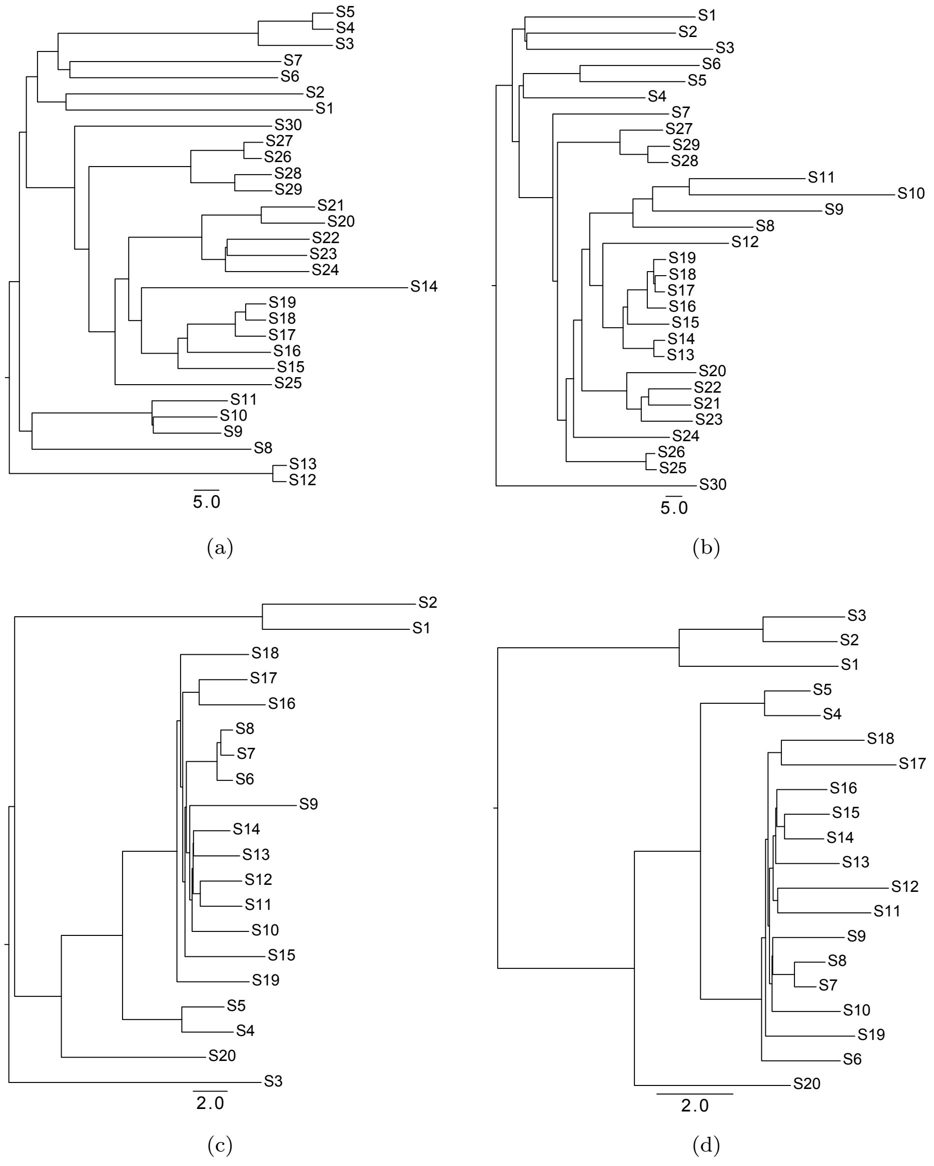

SFigure 1. The species trees used in the simulations.(a)-(b): sampled from bacteria tree; (c)-(d): sampled from mammalia tree.

Gene Tree

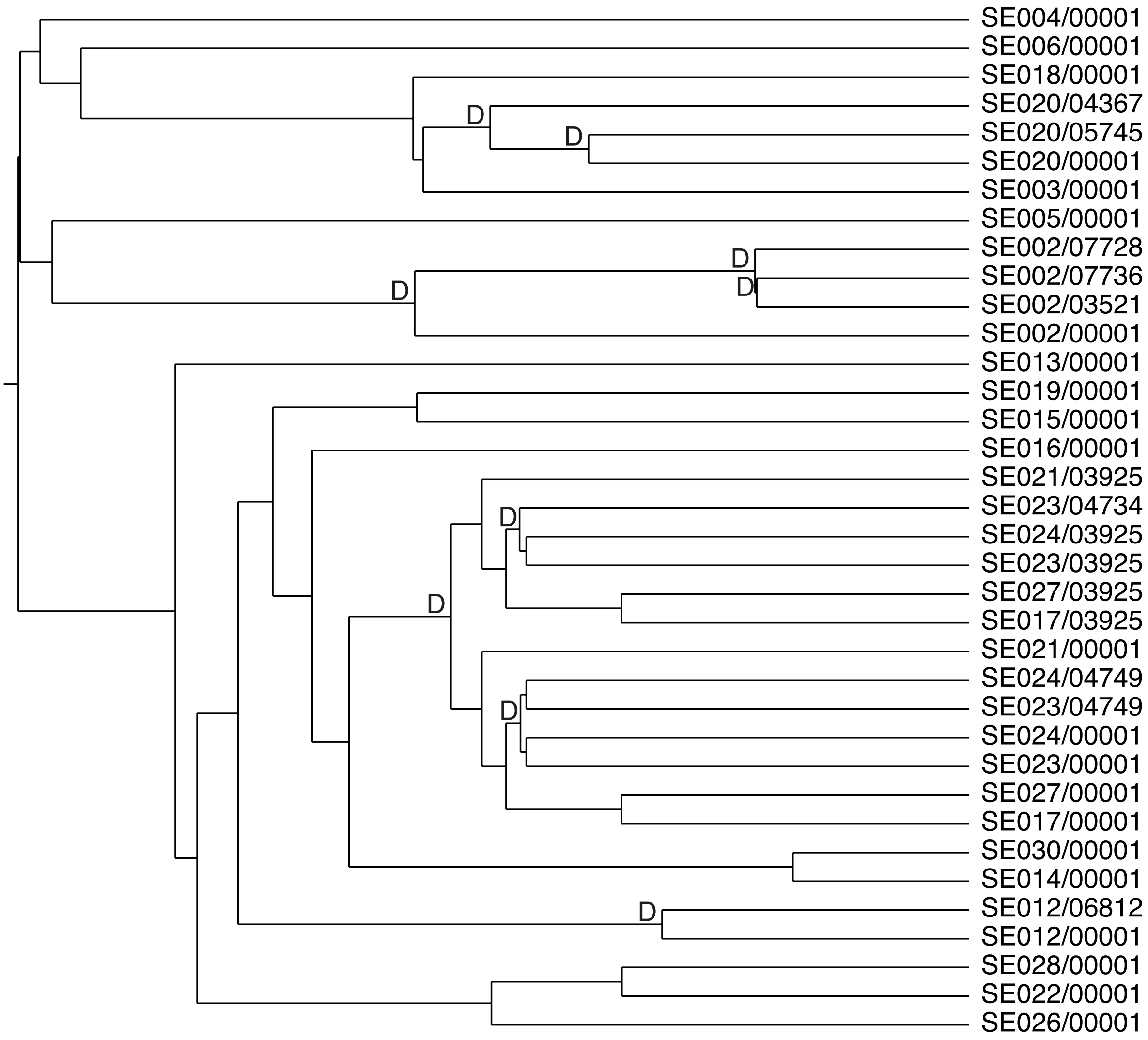

SFigure 2. Example gene tree of a gene family with duplications.

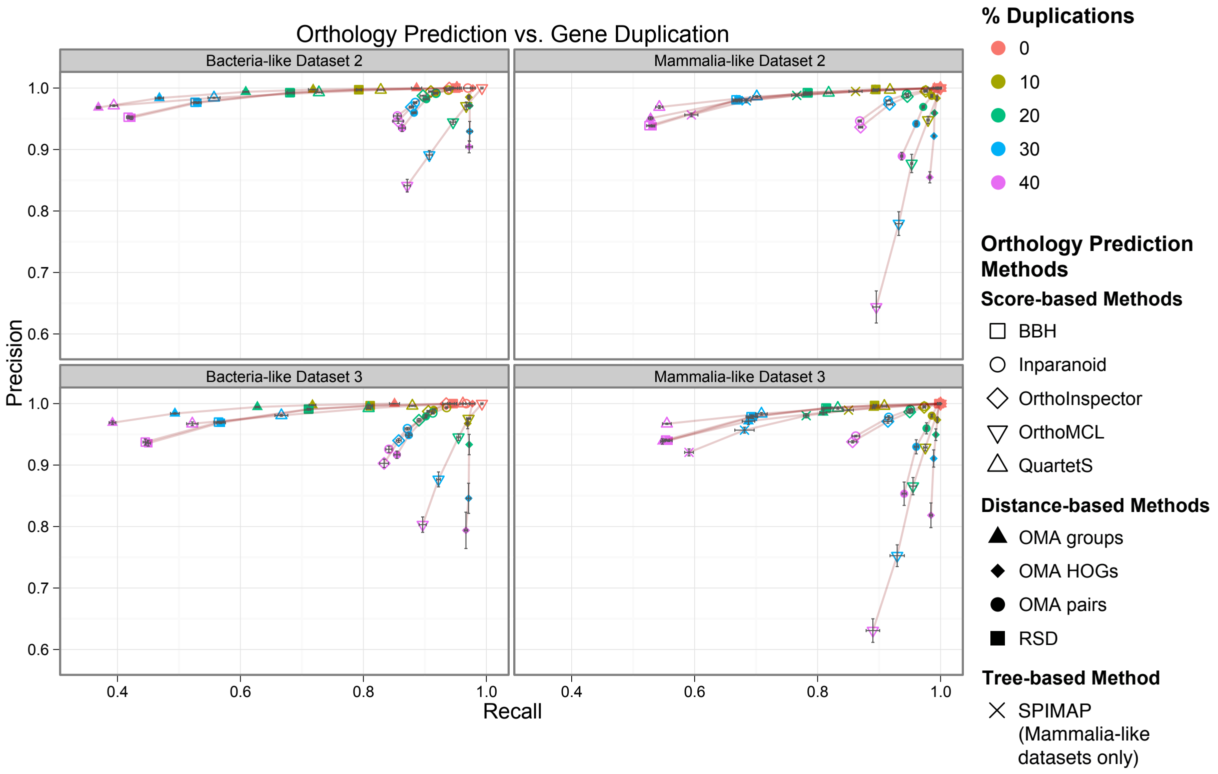

SFigure 3. Results for different proportions of duplications in the replicates not shown in the main article.

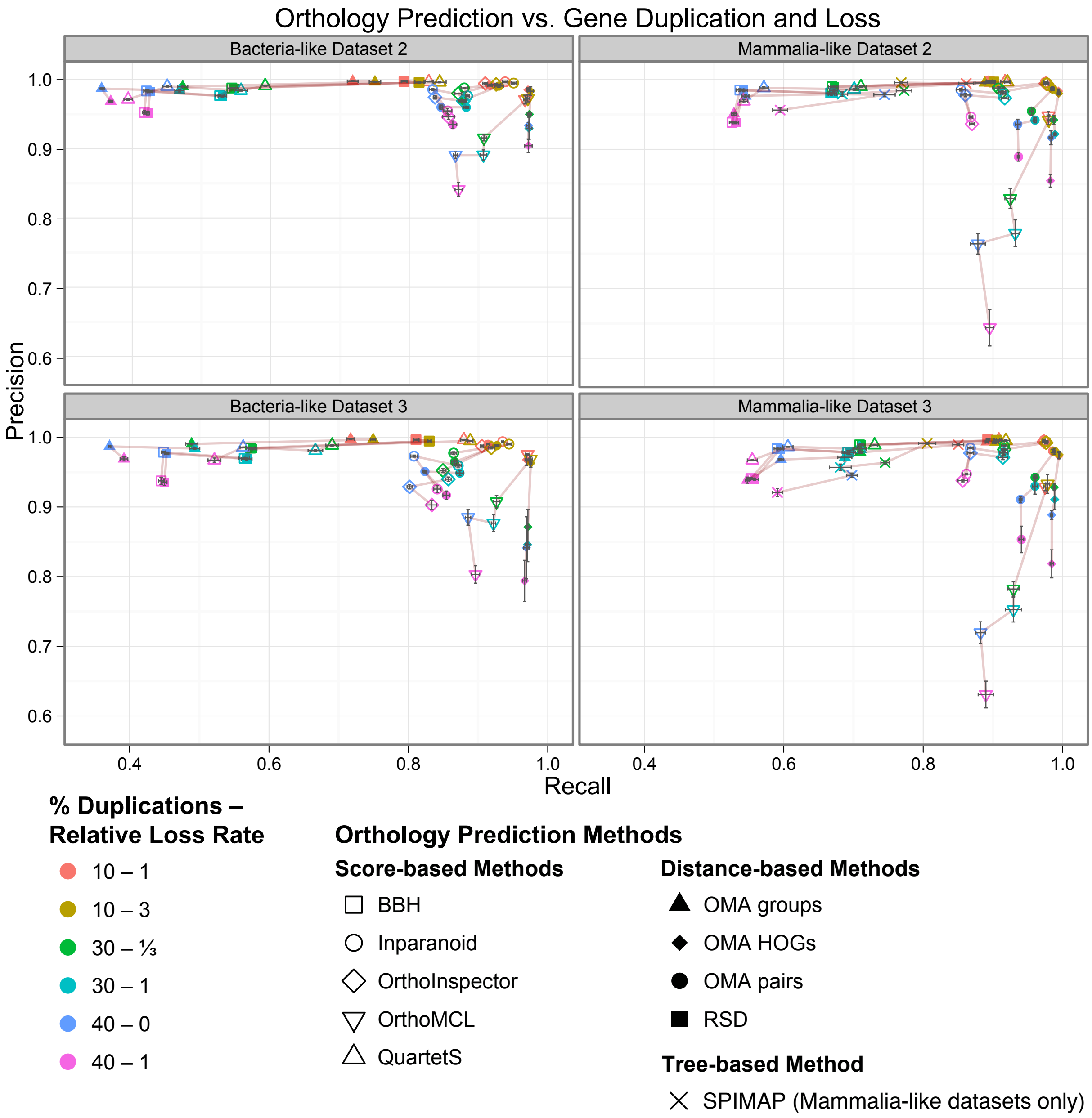

SFigure 4. Results for different proportions of duplications with varying relative loss rates in the replicates not shown in the main article.

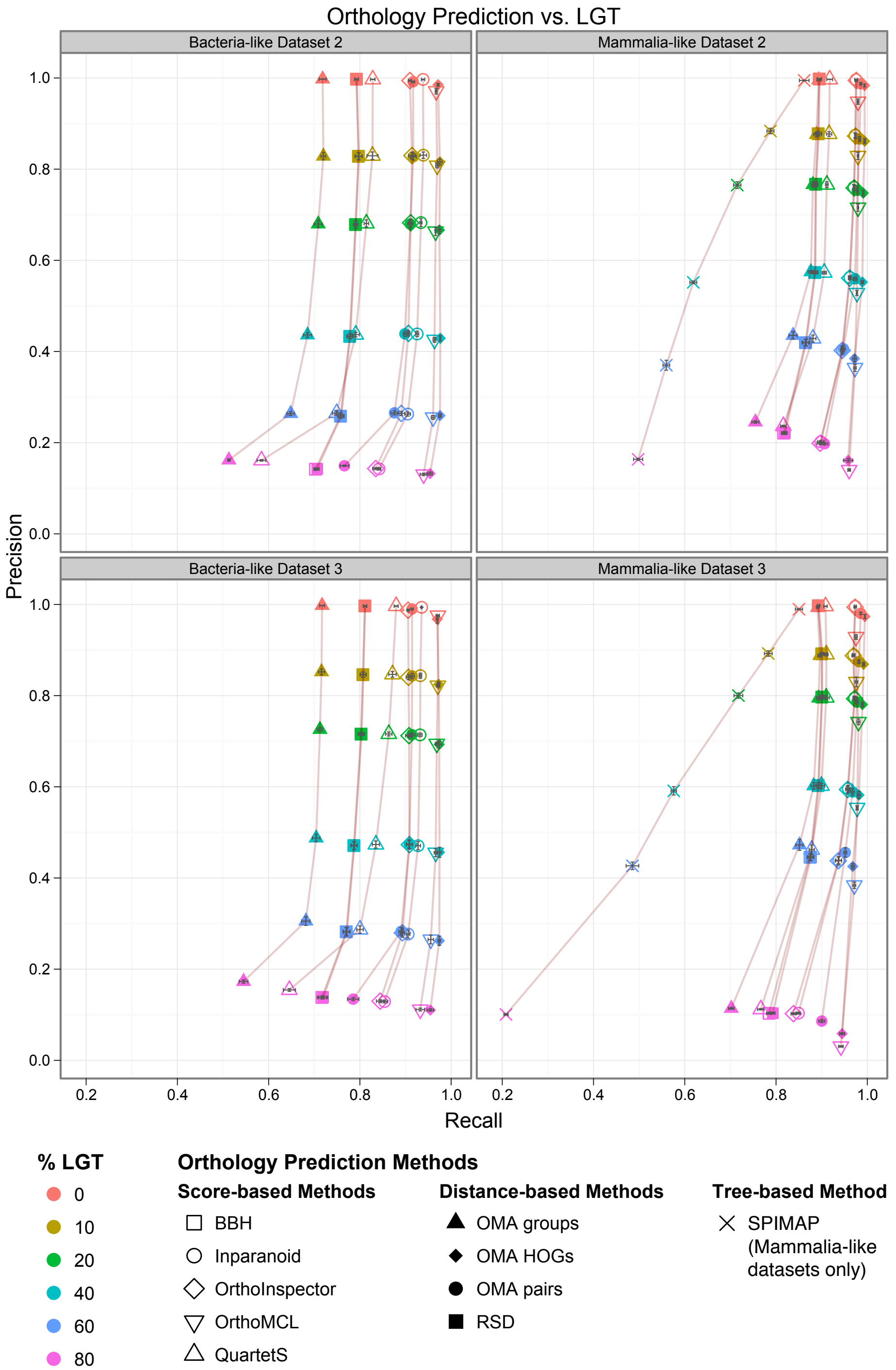

SFigure 5. Results for different proportions of LGT in the replicates not shown in the main article.

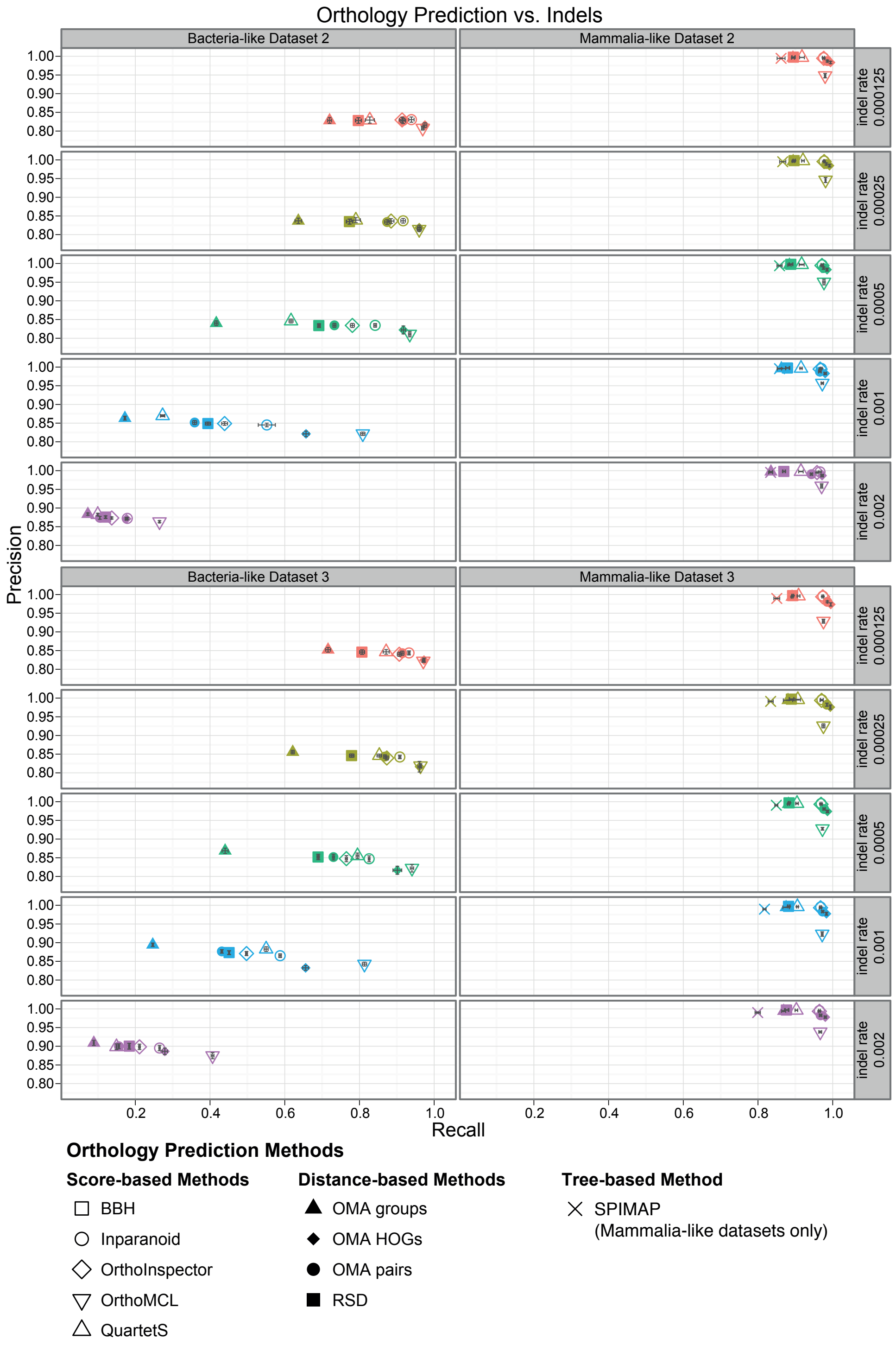

SFigure 6. Results for different insertion and deletion rates.

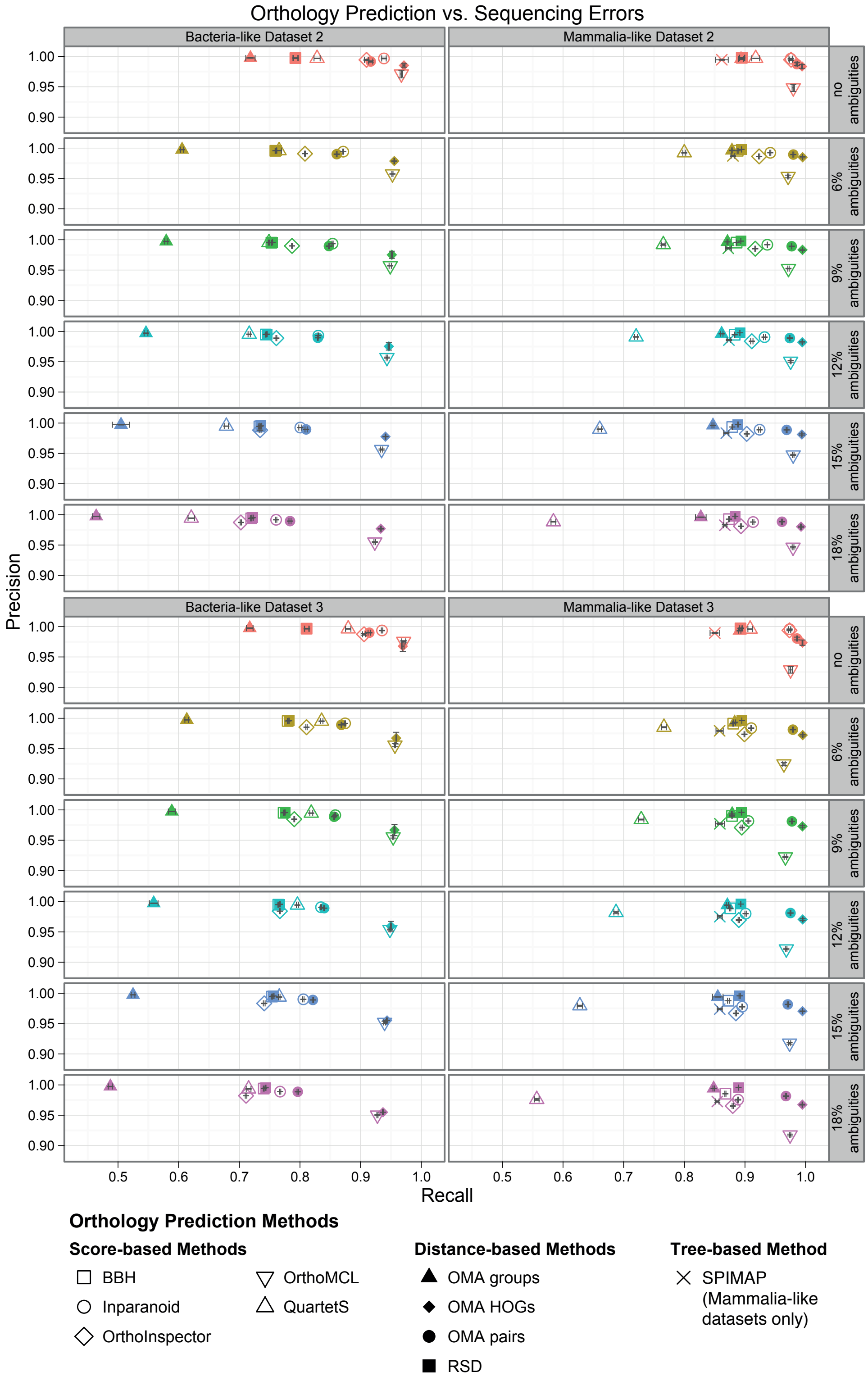

SFigure 7. Results for different proportions of ambiguous characters.

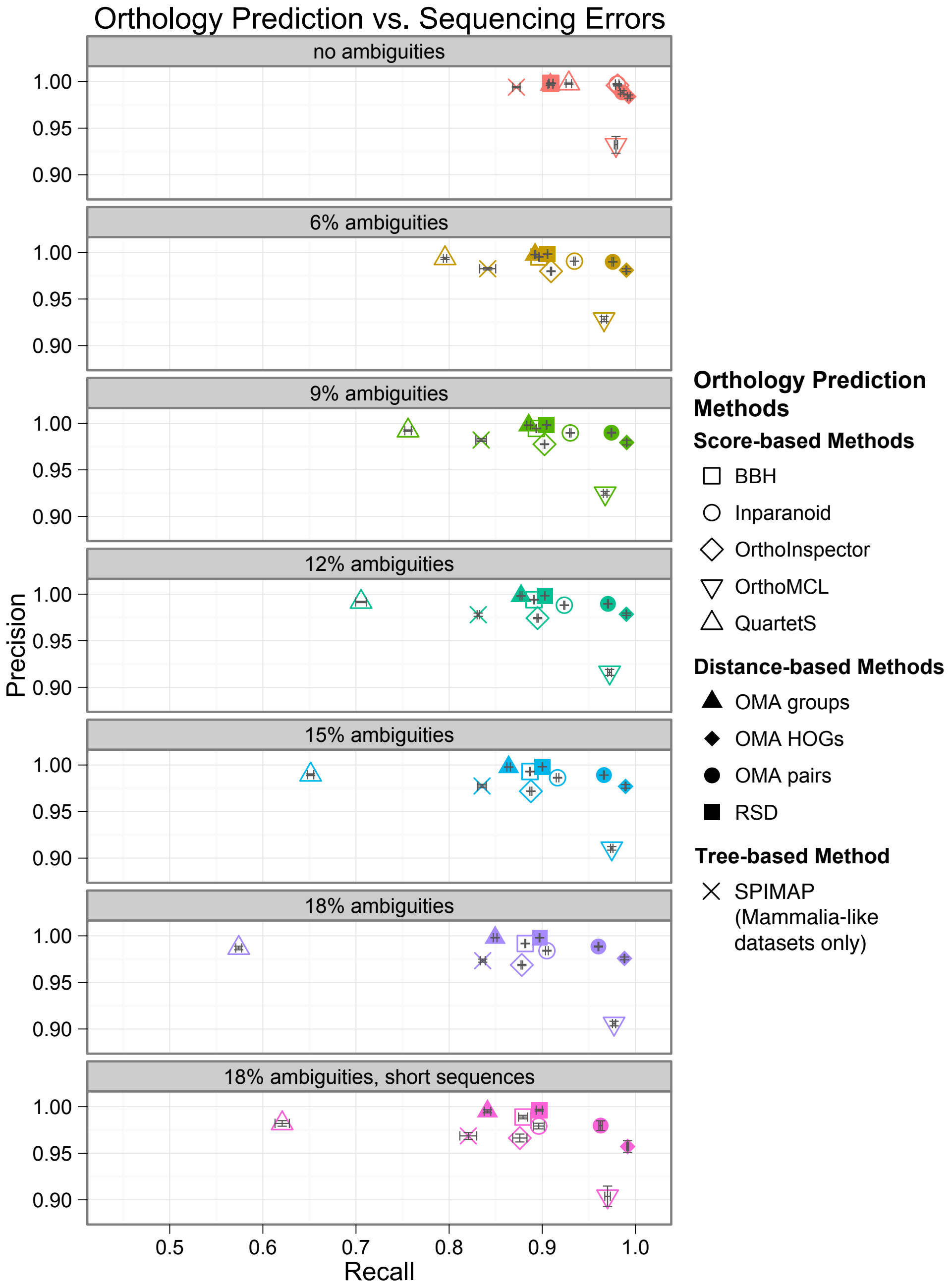

SFigure 8. Results for different proportions of ambiguous characters for mammalia-like dataset 1. We investigated the impact of sequence length on the inference result of QuartetS by creating datasets with shorter sequences (bottom box).
